# Meetings of Societies

**Published:** 1912-03

**Authors:** 


### MEETINGS OF SOCIETIES.

### Bristol Medico-Chirurgical Society.

*December 13th*, 1911.

Mr. C. A. MORTON, President, in the Chair.

Dr. J. MICHELL CLARKE read notes of (1) a case of **Chronic pancreatitis** and (2) a case of **Obscure fever due to a leptothrix blood-infection.** (For the former see p. 25.) The patient with the obscure fever was a shop assistant, aged 23. He was taken ill on September 15th and convalescent on October 21st, 1911. Clinically, the symptoms during the greater part of the illness bore a marked resemblance to those of typhoid fever, with predominance of the nervous manifestations. The leptothrix bacillus was obtained in pure culture on several occasions from different veins and from the cerebro-spinal fluid. This leptothrix has been recognised as pathogenic in one case by Drs. J. Ritchie and Stuart McDonald, and in five other cases met with by the latter during an epidemic of cerebro-spinal meningitis.

Dr. CAREY COOMBS read notes of a case of the **Stokes-Adams syndrome** in a lady, aged 84, running a fatal course in ten days from the onset. Apart from the cerebral symptoms, which consisted of epileptiform and syncopal fits, hallucinations, progressively increasing stupor, and variations in the respiratory rhythm, the chief interest lay in the alterations of cardiac conductivity. In the first phase of the illness there were sudden bouts of complete auriculo-ventricular dissociation, interrupting a sequence which was normal immediately before and after these attacks. In the second phase conductivity was normal, and there were no fits. In the final phase auriculo-ventricular conductivity continuously and progressively declined. There was no autopsy.

Dr. J. O. SYMES read notes on six cases of **Pneumo-thorax.** The first two cases were instances of spontaneous pneumo-thorax following upon violent effort, and skiagrams were shown demonstrating the initial total collapse of the lung and its final restoration to complete recovery. Reference was then made to cases of artificial pneumo-thorax induced, according to Barr's method, for the treatment of obstinate recurring cases of pleural effusion. The results obtained by this method of treatment had been most satisfactory. Three cases of pneumo-thorax were described in order to demonstrate that the lesion might be complete or partial, sudden and painful, or insidious and almost painless. These were all tubercular in origin, and were but little influenced by treatment. Dr. Symes expressed the opinion that the treatment of tubercular pneumo-thorax by incision and drainage was undesirable, even if a considerable quantity of pus were present. The ultimate fate of patients so treated was often gradual failure of health and ultimate death from lardaceous disease. Lantern slides illustrating the various varieties of pneumo-thorax were shown. Dr. COOMBS alluded to two cases of pneumo-thorax due to perforation of the œsophagus which he had seen.—Dr. NIXON pointed out the importance of differentiating hernia through the diaphragm from pneumo-thorax, and cited two cases of this hernia in which diagnosis of pneumo-thorax had been erroneously made.—Dr. CLARKE said that text-books did not lead one to realise that in practice one meets with many cases of pneumo-thorax which had not a tubercular origin. He had always had the thorax drained in cases of pyo-pneumo-thorax.

---

*January* 10*th*, 1912.

Mr. C. A. MORTON, President, in the Chair.

Dr. WALTER C. SWAYNE read a paper on **Operations on the uterus and appendages during pregnancy** (see p. 31). In reply to a question, the speaker said that pregnancy was not a contra-indication for the administration of anæsthetics. Before giving chloroform it should be ascertained that acetone was absent from the urine. Prolonged anæsthesia might have a bad effect on the child.

Dr. C. W. J. BRASHER read a paper on **Pseudo-diphtheria due to diplococcus crassus, with reference to the use of diphtheria Antitoxin.** After referring to the present state of our knowledge of the bacteriology of the throat and nose, and the large number of micro-organisms almost always found there, against which "routine" or "prophylactic" injections of diphtheria antitoxin were of no avail, two cases simulating severe nasal and faucial diphtheria were related. The entire absence of albuminuria was the only symptom by which these cases could be distinguished from true diphtheria. An analysis of the bacteriological reports in the Medical Officer of Health's Office during the last three months of 1911 was given, showing that out of 623 cultures :—

20.45 per cent. showed Klebs-Lœffler bacilli ;
21.1 per cent. showed suspicious forms (*i.e.* Hoffman's bacilli, etc.) ;
0.8 per cent. showed streptococci ;
whilst 76.8 per cent. showed various forms of diplococci.

It was pointed out that these figures were contrary to the usual statistics. The danger of "anaphylaxis" in cases where antitoxin was used in the absence of Klebs-Lœffler bacilli was alluded to. In replying to the remarks of Mr. A. J. Wright, Professor J. Michell Clarke and Dr. W. G. Dickinson, Dr. BRASHER briefly described "diplococcus crassus," and stated that the absence of albuminuria was the only method he could suggest to differentiate clinically this form of pseudo-diphtheria from true diphtheria.

Mr. HEY GROVES read a paper on **Some cases illustrating the difficulty and importance of early diagnosis in acute intestinal obstruction** (see p. 37).

Mr. C. F. WALTERS read notes on a case of **Intussusception in an old man caused by an intestinal polypus,** and showed the specimen.

The PRESIDENT showed a specimen of **Secondary columnar carcinoma of vagina.** The vaginal growth was first detected and then, on examination of the abdomen, there was found to be carcinoma of the pelvic colon. Microscopical sections of both growths were shown.

---

*February 14th,* 1912.

Mr. C. A. MORTON, President, in the Chair.

Dr. E. H. STACK read notes of some **Gall-bladder cases** (see p. 49).

Dr. P. WATSON-WILLIAMS read notes on **Three cases of lateral sinus thrombosis** (patients and specimen shown). Case 1.—Male, aged 37, had been subject to otorrhœa off and on for five years. The temperature was fluctuating between 97° F. and 102° F., but there was no tenderness over the mastoid. Radical mastoid operation was performed. Nine days later the temperature rose to 104° F. The lateral sinus was exposed, and found to be thrombosed. The sinus was opened up nearly to the torcular, and the jugular vein ligatured. The patient improved for a time, but twelve days later became mentally dull, and died forty-eight hours later. The autopsy showed that thrombosis extended a short distance into the opposite left lateral sinus and along the whole length of the longitudinal sinus. There was leptomeningitis of the upper half of the cerebral hemispheres. There had been a remarkable extension of thrombosis in this case, unaccompanied by definite symptoms suggesting that condition. Case 2.—The patient, a boy of 12, had severe headache, vomiting, and tenderness behind the left ear. Temperature at night 102° F. A large mastoid antrum full of pus was opened, and some pus evacuated from an extra-dural abscess over the lateral sinus. There was no evidence of sinus thrombosis of the jugular. Case 3.—Male, aged 24. Ten days' pain in right ear, headache and rigors. Temperature 102° F. to 103° F. Large right aural polypus present. The mastoid antrum contained pus, and there was pus over the lateral sinus. After disinfection a hypodermic needle was passed into the sinus, and some clot blocking the needle withdrawn. A second deeper puncture extracted fluid blood, and on culture the blood yielded staphylococci and a few streptococci. The jugular vein was not ligatured. Uneventful recovery.—Mr. Lacy Firth said that Case 1 seemed to show the importance of clearing out all clot towards the torcular end of the sinus, and establishing free bleeding whenever it was feasable. His practice was to ligature the jugular vein in almost all cases of thrombosis of the lateral sinus, and to do that before removing the septic material from the sinus. In Case 3 he thought there existed a little doubt as to whether or not the blood obtained from the sinus had been contaminated during its removal through incomplete disinfection of the punctured spots.

Dr. A. Rendle Short read a paper on **End-results of operations for internal derangements of the knee-joint** (see p. 52).—Mr. Hey Groves, Dr. Nixon, Dr. Donaldson, and the President discussed the paper.

Dr. C. E. K. Herapath read notes of **A case of partial heart-block occurring in acute rheumatism.** A boy aged 16 was admitted to the Bristol Royal Infirmary on October 30th, 1911, suffering from acute rheumatism of four days' duration. He had no previous illness. The size of the heart and the heart-sounds were normal on admission, but pauses were noticed during which no heart-sounds were heard. These were proved by tracings of the jugular and arterial pulses to be due to partial heart-block. This continued for eight days, when the tracings became normal. On November 23rd the patient had a relapse. Eight days after this he developed another attack of partial heart-block, which lasted for thirteen days. The subsequent tracings were normal. Defects in conductivity in the bundle of His have been shown to be increased by vagal stimulation, either directly or indirectly by drugs, such as digitalis. A good number of cases of heart-block have been shown to depend on vagal stimulation. In this case neither direct stimulation nor indirect stimulation by digitalis caused any increase of A—C interval. It is suggested, therefore, that the heart-block depended not on vagal influence but on actual rheumatic inflammation of the bundle of His.—Dr. Carey Coombs alluded to the rarity of heart-block in rheumatic fever. The lesion might be focal or diffuse in the muscle fibres. A focal lesion must be present to interrupt conductivity. A small inflammatory nodule might have formed in the bundle of His. The majority of instances of heart-block had been in males, and in patients of a later age than in the case now reported.—Dr. Nixon spoke of the difficulty of taking tracings from patients with acute rheumatism. The case showed how transitory the condition might be.

J. Lacy Firth.
J. A. Nixon, *Hon. Sec.*

